# Accelerated partial breast irradiation using robotic radiotherapy: a dosimetric comparison with tomotherapy and three-dimensional conformal radiotherapy

**DOI:** 10.1186/s13014-016-0607-9

**Published:** 2016-02-27

**Authors:** Erwann Rault, Thomas Lacornerie, Hong-Phuong Dang, Frederik Crop, Eric Lartigau, Nick Reynaert, David Pasquier

**Affiliations:** Academic Department of Radiation Oncology, Centre Oscar Lambret, 3 rue Frédéric Combemale, BP 307, 59020 LILLE Cedex, France; CRIStAL, UMR CNRS 9189, Villeneuve d’Ascq, France

**Keywords:** Accelerated partial breast irradiation, Robotic stereotactic radiotherapy, Surgical clips tracking

## Abstract

**Background:**

Accelerated partial breast irradiation (APBI) is a new breast treatment modality aiming to reduce treatment time using hypo fractionation. Compared to conventional whole breast irradiation that takes 5 to 6 weeks, APBI is reported to induce worse cosmetic outcomes both when using three-dimensional conformal radiotherapy (3D-CRT) and intensity-modulated radiotherapy (IMRT). These late normal tissue effects may be attributed to the dose volume effect because a large portion of the non-target breast tissue volume (NTBTV) receives a high dose. In the context of APBI, non-coplanar beams could spare the NTBTV more efficiently. This study evaluates the dosimetric benefit of using the Cyberknife (CK) for APBI in comparison to IMRT (Tomotherapy) and three dimensional conformal radiotherapy (3D-CRT).

**Methods:**

The possibility of using surgical clips, implanted during surgery, to track target movements is investigated first. A phantom of a female thorax was designed in-house using the measurements of 20 patients. Surgical clips of different sizes were inserted inside the breast. A treatment plan was delivered to the mobile and immobile phantom. The motion compensation accuracy was evaluated using three radiochromic films inserted inside the breast. Three dimensional conformal radiotherapy (3D-CRT), Tomotherapy (TOMO) and CK treatment plans were calculated for 10 consecutive patients who received APBI in Lille. To ensure a fair comparison of the three techniques, margins applied to the CTV were set to 10 mm. However, a second CK plan was prepared using 3 mm margins to evaluate the benefits of motion compensation.

**Results:**

Only the larger clips (VITALITEC Medium-Large) could be tracked inside the larger breast (all gamma indices below 1 for 1 % of the maximum dose and 1 mm). All techniques meet the guidelines defined in the NSABP/RTOG and SHARE protocols. As the applied dose volume constraints are very strong, insignificant dosimetric differences exist between techniques regarding the PTV coverage and the sparing of the lung and heart. However, the CK may be used to reduce high doses received by the NTBTV more efficiently.

**Conclusions:**

Robotic stereotactic radiotherapy may be used for APBI to more efficiently spare the NTBTV and improve cosmetic results of APBI.

## Introduction

Breast cancer is the most common cancer in women, representing approximately 25 % of all cancers in women [1]. Conventional treatments range from surgery to radiotherapy of the whole breast. The recommended dose for the whole breast varies between 40 and 50 Gy delivered in 3 to 5 weeks followed by 16 Gy to the resection margins in younger women. Accelerated partial breast irradiation (APBI) is a new treatment modality aiming to reduce treatment time using fraction doses higher than conventional fractionation. The total dose is between 38 and 40 Gy in 10 fractions delivered in 5 days. As opposed to whole breast irradiation (WBI), the dose is given only to the resection volume. Multiple techniques have been tried, including intraoperative radiotherapy, brachytherapy and conformal radiotherapy. APBI using conformal radiotherapy is investigated in several randomized studies around the world, including a French multicenter phase III trial. Prescribed doses are identical in RTOG and French trials (NSABP/RTOG protocol, SHARE protocol [2]). Cosmetic results after APBI seem controversial [3]. Worse cosmetic outcomes have been reported using three-dimensional conformal radiotherapy (3D-CRT) [4] as well as intensity-modulated radiotherapy (IMRT) [5] for APBI. In a randomized trial, 3D-CRT APBI increased rates of adverse cosmesis and late radiation toxicity compared with standard WBI [6]. These late normal tissue effects may be attributed to the dose volume effect [7] because a large portion of the non-target breast tissue volume (NTBTV) receives a high dose.

The CyberKnife (Accuray Incorporated, Sunnyvale CA, USA) [8, 9] is a frameless robotic radiosurgery system. In the context of APBI, the Cyberknife could spare the NTBTV more efficiently because of the combination of non-coplanar fields and tracking of the target volume. In a publication from Vermeulen et al. [10], excellent/good cosmetic outcomes were described after a follow-up period of 12 months for 9 patients who received APBI using the CyberKnife. A dosimetric investigation of APBI using the CyberKnife suggests the same results [11]. Our study proposes to investigate the effect of non-coplanar beams for APBI. This effect is first evaluated comparing Cyberknife, IMRT (Tomotherapy) and 3D-CRT treatment plans calculated for 10 patients using the same PTV margins. The feasibility and benefit of tracking the target volume are evaluated in a second part. Synchrony, the respiratory tracking system provided with the Cyberknife, uses diagnostic x-ray images of fiducials implanted in the target volume to correlate the position of the target volume to the position of external markers. This model enables the linear accelerator to continuously track the motion of the target volume regarding the position of external markers. The possibility of using surgical clips that are implanted during surgery to track target movements is investigated here to avoid an additional invasive procedure. The benefit of tracking using the Cyberknife is evaluated at last and a Cyberknife treatment plan is calculated using reduced PTV margins.

## Materials and methods

### A. Treatment planning

Ten consecutive patients were selected for this study. This study was approved by the scientific board of the multidisciplinary breast tumor institutional group. These patients received 3D-CRT APBI in the Oscar Lambret Cancer Center in Lille, France between December 2010 and October 2012. A radiotherapy treatment planning CT was acquired for each patient. Patients were positioned in the supine position using a breast board. The CT started at or above the mandible and extended several centimeters below the inferior limit of the breast. According to our 3D-CRT protocol, the pixel size was set to 1 mm in the transverse plane and 3 mm in the longitudinal direction. The target and organs at risk delineation were realized according to SHARE and NSABP/RTOG 0413 guidelines. The following structures were contoured: CTV, NTBTV, contralateral breast, heart, homolateral lung and contralateral lung. Volume expansion was limited to exclude pectoralis muscles, the chest wall and the first 3 mm beneath the skin.

Three radiotherapy treatment plans were calculated for each patient using 10 mm margins between the CTV and PTV: 3D-CRT, Tomotherapy (TOMO) and CyberKnife (CK). TOMO and CK plans were only computed for technique comparison purposes and may not be relevant for treatment. A second Cyberknife plan was computed using 3 mm PTV margin (CK_RM_) to take into account the motion compensation abilities of the CyberKnife (Synchrony). 3 mm is the margin used in our institution for synchrony liver treatments. The 3D-CRT plans were designed using ONCENTRA MASTERPLAN (Elekta Inc., Stockholm, Sweden). Three beams were primarily used: two tangential photon beams (6 or 20 MV depending on size and shape of the breast) and a direct electron beam whose energy depends on the depth of the tumor. A dose of 40 Gy was prescribed to the PTV, with 39 Gy covering at least 95 % of the PTV. Dose calculations were made using the collapsed cone algorithm and a 3 mm dose grid. Tomotherapy plans were calculated using a Tomotherapy planning station (version 4.2). A directional block was used to prevent the use of opposed beams. The final dose was calculated using the convolution/superposition algorithm based on the collapsed cone approach [12] in fine resolution (pixel size of 1 mm). CyberKnife plans were optimized using both Multiplan (CyberKnife planning system version 4.6.0) and the dynamic collimator (IRIS). Collimator sizes were adapted to the target volume to optimize conformality while maintaining reasonable treatment times (40 min). Final dosimetric calculations were computed using the Monte Carlo algorithm in a 2 mm dose grid and a statistical uncertainty of 2 %. All plans satisfy the constraints (Table 1) defined in the SHARE and NSABP/RTOG protocols. In the case conflicting constraints, the most restrictive one was chosen.

**Table 1:** Constraints given by the SHARE and NSABP/RTOG protocols used to optimize all treatment plans

PTV	D_max_ < 44 Gy
	D_99%_ ≥ 38Gy
	D_95%_ ou D_90%_ ≥ 40 Gy
Homolateral lung	V_20Gy_ < 1.3 %
	V_12Gy_ < 15 %
	V_10Gy_ < 5.7 %
	V_5Gy_ < 8 %
Contralateral lung	V_20Gy_ < 1 %
	V_10Gy_ < 2 %
	V_5Gy_ < 3 %
	V_2Gy_ < 15 %
Heart	V_20Gy_ < 0.5 %
	V_10Gy_ < 1 %
	V_5Gy_ < 4.1 %
	V_2Gy_ < 5 % for left lesions
	V_2Gy_ < 40 % for right lesions
Contralateral breast	D_max_ < 3 % of prescribed dose (D_max_ < 1.3 Gy)
NTBV	V_20Gy_ < 50 %
	V_18Gy_ <60 %

### B. Dosimetric parameters for plan comparison

For each treatment plan, the mean dose delivered to 2 % (D_2%_) and 98 % (D_98%_) of the PTV were reported. The homogeneity index (HI) and the Dice similarity coefficient (DSC) were calculated using the definitions from *ICRU report 83* [13]. The homogeneity index is defined as:

$$ HI=\frac{D_{2\%}-{D}_{98\%}}{D_{50\%}} $$

The Dice similarity index is defined as the ratio of the intersection between the treated volume (TV) and the PTV over the sum of these volumes:

$$ DSC=\frac{2\ast \left(TV\cap \kern0.15em PTV\right)}{TV+PTV} $$

Following the recommendations from *ICRU report 86*, the target volume is defined as the volume receiving at least 98 % of the maximum dose.

Doses delivered to the OAR were compared using dose volume histograms (DVH) and organ specific dosimetric data: the mean dose (D_mean_), the near-maximum dose (D_2%_), the volumes receiving 18 (V_18Gy_) and 20 Gy (V_20Gy_) for the NTBTV, the volumes receiving 5 (V_5Gy_), 10 (V_10Gy_) and 20 Gy (V_20Gy_) for the homolateral lung, the volumes receiving 2 (V_2Gy_) and 5 Gy (V_5Gy_) for the heart and the near-maximum dose (D_2%_) for the contralateral breast. The dose delivered to the heart was calculated only in the five patients with left-sided breast cancer. In this study, we compared the CyberKnife to other techniques; we did not compare these techniques with each other. Significant differences between techniques were enhanced using the Wilcoxon signed-rank test (significance threshold *p* < 0.05).

### C. Surgical clip tracking

Using Synchrony to track respiratory motions could reduce PTV margins from 10 to 3 mm and thus reduce the dose delivered to the NTBTV. However, fiducial markers are required to follow target volume movements inside the patient. Implantation of fiducial markers is an additional invasive act for a patient. Titanium surgical clips are used in our institution to delineate the excision volume after surgery. If these clips would be visible for the tracking system of the CyberKnife, the implantation of fiducial markers would no longer be required.

**Fig. 1:**
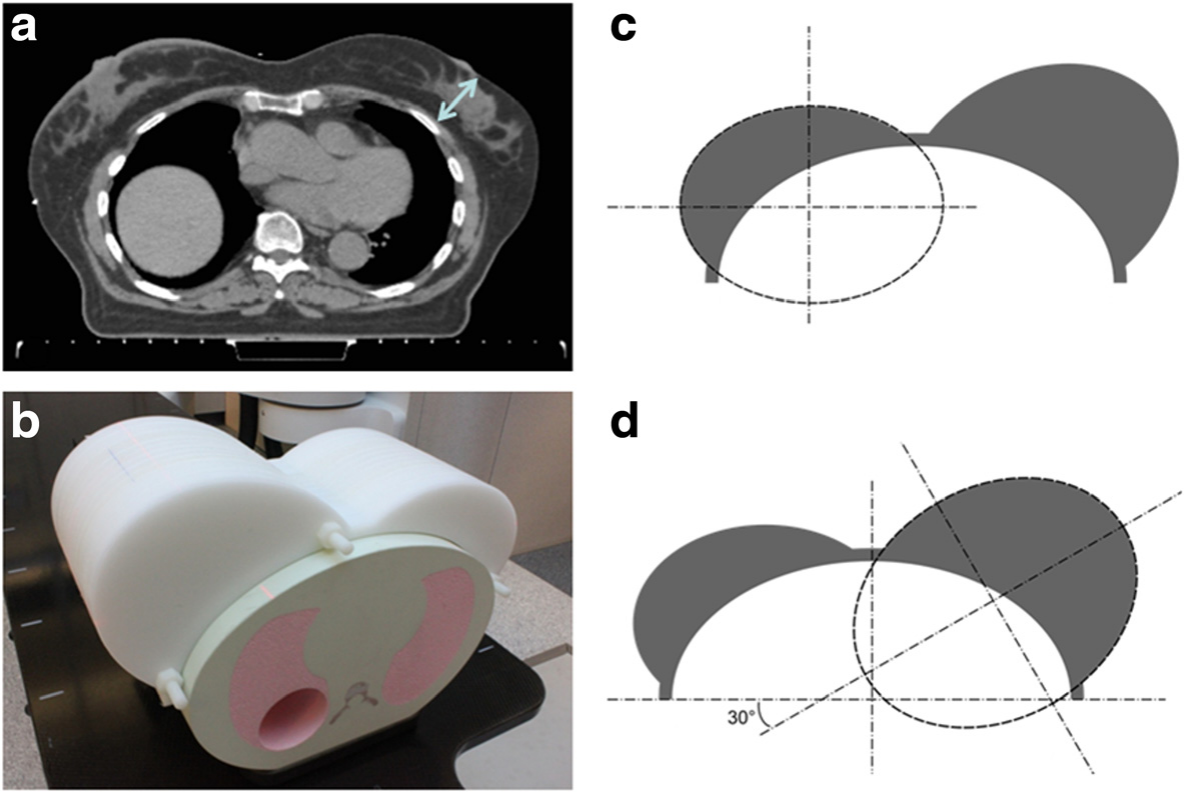
Breast phantom: thickness of the breast measured between the chest wall and the skin on 30 patients’ CT (**a**), breast phantom geometry with two breasts of different dimensions (**c** and **d**) and breast phantom positioned on the Xsight lung phantom (**b**)

A realistic breast phantom was designed to study surgical clip tracking. The new phantom can be used in combination with the Xsight lung thorax (CIRS Inc., Norfolk, USA) phantom proposed for the CyberKnife. As shown in Fig. 1a, breast size was measured in the middle of the breast between the skin and the chest wall for 30 consecutive patients treated for breast cancer. Two breast sizes were used: the medium size (55.4 mm) for the right breast and the largest size (106 mm) for the left breast. Figure 1b shows the breast phantom positioned on top of the Xsight lung phantom. The phantom geometry was described using two ellipses (Fig. 1c and d). The breast phantom is made of natural polyethylene (0.97 g.cm^−3^) and composed of 10 slices (5 external slices of 20-mm width and 5 central slices of 10-mm width). This material is similar to breast tissues in the energy range of photons used for diagnostic radiology. Radiochromic films were inserted between the slices of the phantom to record the dose distribution. All radiochromic films were taken from the same batch. This batch was calibrated in dose response a week before the experiments using the red and blue channels. Exposed films were scanned 14 h after irradiation at 72 dpi in 48-bit RGB transmission mode on an Epson Expression 10000 XL.

Three titanium surgical clips of different sizes were tested: VITALITEC 6 Small-Medium (Vitalitec International, Inc., Plymouth, USA), LIGACLIP EXTRA medium (ETHICON Inc., Cornelia, USA) and VITALITEC Medium-Large. Four cavities were bored inside the phantom in each breast to insert the clips. A CyberKnife treatment plan was created to deliver 3.8 Gy in an irregular volume surrounding the clips. Clips of each size were inserted inside the phantom, and the treatment plan was delivered to both an immobile and mobile phantom (with and without Synchrony). In each case, three Gafchromic films were inserted inside the phantom (central slices containing the clips) to record the dose distribution in three parallel plans. Phantom movement was longitudinal with frequencies and amplitudes (3 cm) close to the values of human breathing [14, 15].

The gamma index [16] was chosen to quantify the agreement between dose distributions measured with or without Synchrony and the dose distributions measured without any movement of the phantom (gold standard). Gamma indices were calculated using an in-house Matlab routine. Films were registered using three points corresponding to spatial markers at the surface of the breast phantom and drawn on the Gafchromic films. Gamma index limits were set to 1 mm, 1 % (3 Cgy) corresponding to the best results we could obtain.

## Results

### A. Surgical clip tracking

**Fig. 2:**
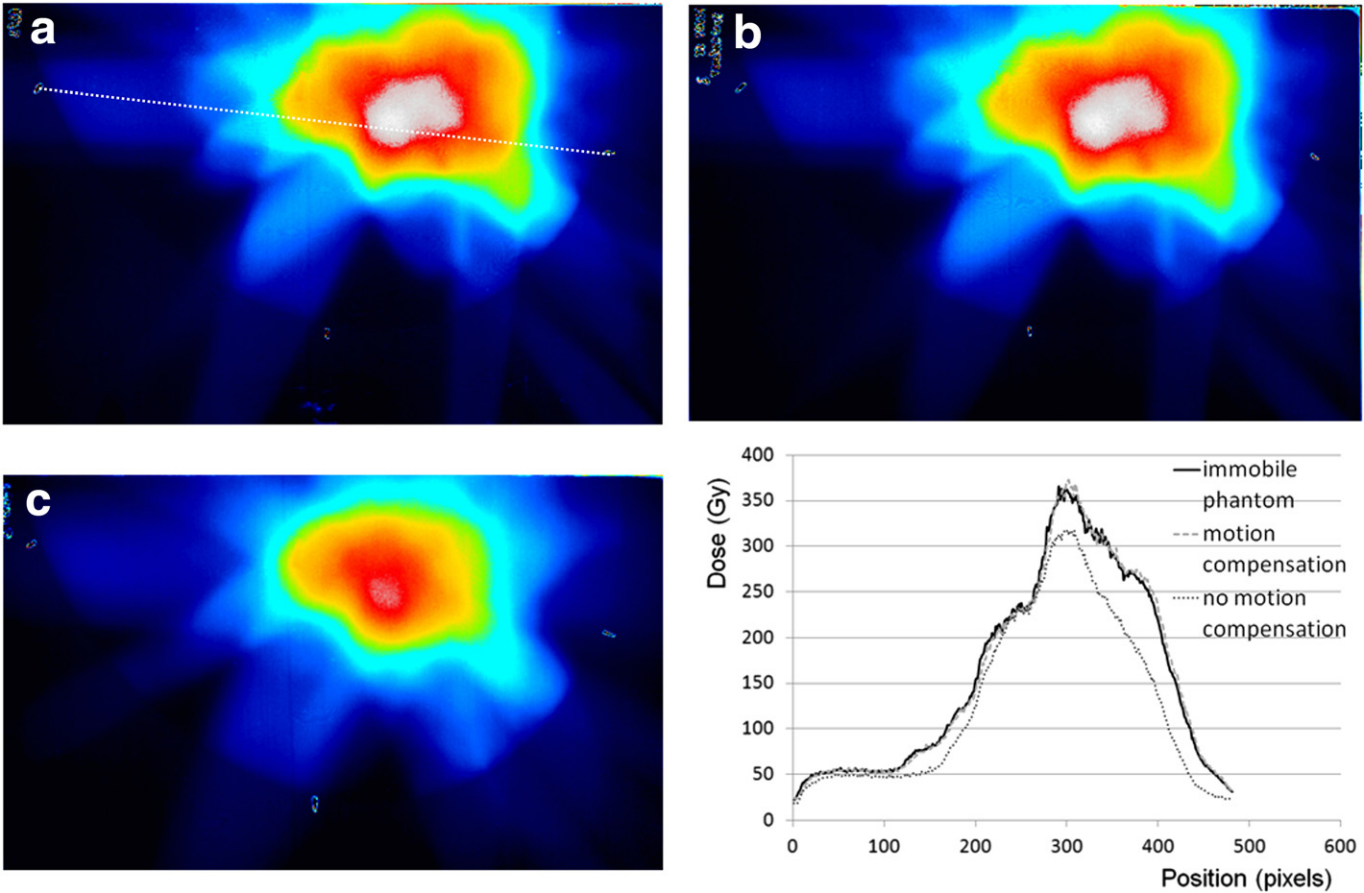
Dose distributions measured inside the left breast for an immobile (**a**) and a mobile phantom with (**b**) or without (**c**) motion compensation. Dose profiles recorded along the white dotted line drawn on figure a are displayed on the left bottom side of the figure

Only the larger clips (VITALITEC Medium-Large) could be tracked using Synchrony inside the left breast (larger breast, worst case scenario). The dose distributions displayed in Fig. 2 are measured using Gafchromic EBT3 films inside the left breast. Only gamma indices calculated inside the breast were considered. The results show that dose distributions agree very well when Synchrony is used for respiratory motion compensation (all gamma indices below 1 for 1 mm and 3 cGy).

### B. Dosimetric comparison

**Fig. 3:**
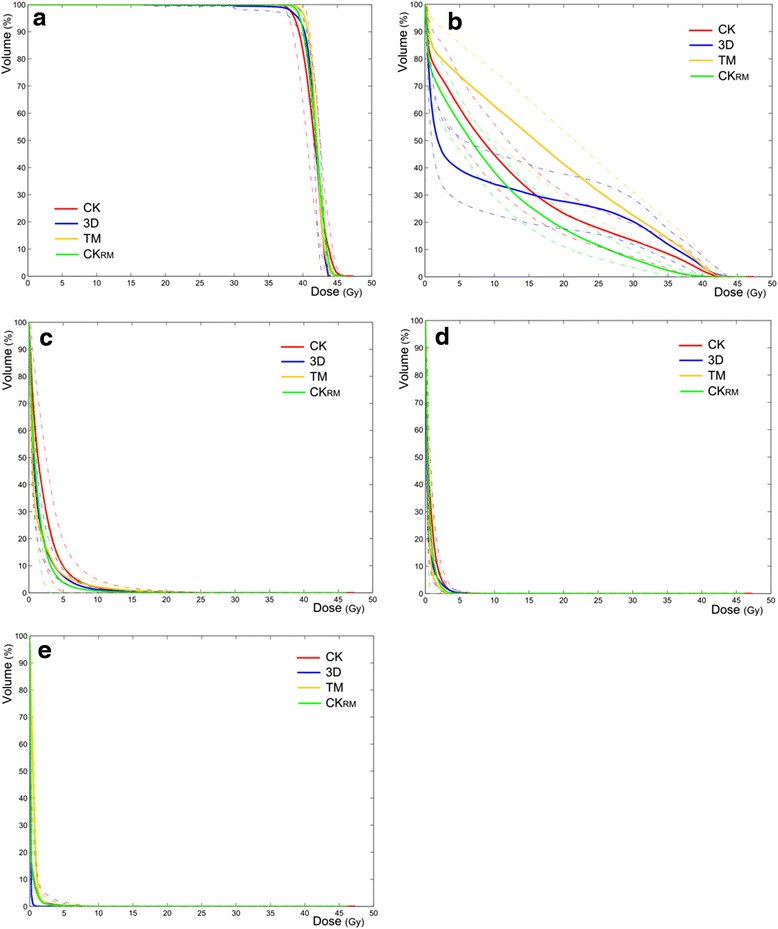
Mean DVH data for PTV (**a**), NTBTV (**b**), homolateral lung (**c**), heart (**d**) and contralateral breast (**e**). PTV: planning target volume; NTBTV: non-target breast tissue volume. 3D: 3D-conformal radiation therapy, TM: Tomotherapy, CK: CyberKnife, CKRM: CyberKnife with reduced margins

Table 2 shows mean dosimetric data computed for each treatment modality. Significant differences between CK_RM_ and the other techniques are highlighted in bold font (*p*-values < 0.05). Corresponding mean DVHs are displayed in Fig. 3 for all treatment modalities.

**Table 2:** Comparison of PTV, heart, lung and NTBTV dosimetric data for 3D-CRT, tomotherapy (TOMO), CyberKnife (CK) and CyberKnife with reduced margins (CKRM). CK_RM_ data are written in bold letters when there are significant differences between CK_RM_ and at least one other technique (only *p*-values < 0.05 are specified)

	3D-CRT	TOMO	CK	CK_RM_	*P value CK-RM vs RC3D*	*P value CK-RM vs TM*	*P value CK-RM vs CK*
PTV							
HI	0.132 ± 0.079	0.097 ± 0.016	0.123 ± 0.01	0.121 ± 0.01			
DSC	0.805 ± 0.047	0.769 ± 0.053	0.850 ± 0.04	0.858 ± 0.03			
D_98%_ (Gy)D_2%_ (Gy)	37.5 ± 3.1	39.8 ± 0.5	38.9 ± 0.9	39.0 ± 0.3			
	43.1 ± 0.5	43.8 ± 0.4	44.1 ± 1.1	44.1 ± 0.3			
NTBTV							
D_mean_ (Gy)	11.3 ± 3.7	17.3 ± 4.0	12.3 ± 3.0	**9.9 ± 2.0**		**0.002**	**0.002**
D_2%_ (Gy)	40.5 ± 1.2	40.3 ± 1.2	39.4 ± 1.5	**34.6 ± 2.6**	**0.0020**	**0.002**	**0.002**
V_18Gy_ (%)	28.6 ± 10.2	45.5 ± 12.5	26.4 ± 8.2	**20.7 ± 7.1**		**0.002**	**0.0039**
V_20Gy_ (%)	27.7 ± 10.1	41.3 ± 12.1	23.3 ± 7.8	**17.6 ± 6.5**	**0.0195**	**0.0059**	**0.0039**
Homolateral Lung							
V_5Gy_ (%)	6.2 ± 2.3	6.6 ± 3.1	9.8 ± 9.5	**3.8 ± 6.1**			**0.002**
V_10Gy_ (%)	1.2 ± 0.5	2.3 ± 1.6	2.0 ± 2.9	**0.7 ± 1.5**		**0.0059**	**0.002**
V_20Gy_ (%)	0.2 ± 0.2	0.3 ± 0.5	0.2 ± 0.4	**0.0**	**0.0078**	**0.0156**	**0.0312**
Heart							
V_2Gy_ (%)	4.7 ± 3.4	2.2 ± 3.1	7.5 ± 6.7	3.9 ± 7.6			
V_5Gy_ (%)	0.2 ± 0.4	0.0 ± 0.0	0.3 ± 0.7	0.0 ± 0.0			
Contralateral Breast							
D_2%_ (Gy)	0.3 ± 0.2	1.7 ± 1.0	1.3 ± 2.0	1.3 ± 1.8			

*PTV* planning target volume, *NTBTV* non-target breast tissue volume, *3D-CRT* three-dimensional conformal radiotherapy

For PTV coverage, the DVHs displayed in Fig. 3a for all treatment modalities are very close. The mean HI is between 0.132 (3D) and 0.0097 (TOMO) and the mean DSC is between 0.769 (TOMO) and 0.858 Gy (CK_RM_). PTV near-min doses (D_98%_) range from 37.5 (3D-CRT) to 39.0 (CK_RM_) and near-maximum doses range from 43.1 (3D-CRT) to 44.1 Gy (CK and CK_RM_) (*p* > 0.05).

As shown in Fig. 3b, DVHs of the NTBTV show significant differences between techniques. The use of non-coplanar beams (CK and CK_RM_) reduces high doses to the NTBTV. Significant differences were found between CK_RM_ and all other techniques for the near-maximum dose (D_2%_) and the volume receiving 20 Gy (V_20Gy_) of the NTBTV. Mean doses to the NTBTV vary from 17.3 (TOMO) to 9.9 Gy (CK_RM_). V18Gy ranges from 20.7 (CK_RM_) to 45.5 % (TOMO) (Table 2).

Dosimetric data are very close for the homolateral lung, heart and contralateral breast. These results are displayed using DVHs in Fig. 3c, d and e. Significant differences were found for the volume of homolateral lung receiving 20 Gy (V_20Gy_). Values range from 0.0 Gy for CK_RM_ to 0.3 Gy for TOMO.

## Discussion

In the literature, cosmetic results of APBI remain controversial. The interim NSABP B-39/RTOG 0413 randomized trial report showed a low and acceptable toxicity rate [17]. Some adverse cosmetic effects may be attributed to the dose volume effect because a large portion of the NTBTV receives a high dose [7]. In Leonard et al. [18], the relative volume of breast tissue receiving 5 to 100 % (V_5_-V_100_) of the prescribed dose was associated with a risk of subcutaneous fibrosis, and the volume receiving 50 to 100 % (V_50_-V_100_) was associated with fair/poor cosmesis in 80 patients. In a Moffitt Cancer Center series of 94 patients who were treated according to RTOG 0413 guidelines, increasing the percentage of ipsilateral breast volume receiving more than 50 % of the prescription dose (V_50%_) was correlated with less than excellent cosmesis (*p* < 0.001) with a threshold V_50%_ of 40 % [19]. In Olivotto et al. [6], APBI increased rates of adverse cosmesis and late radiation toxicity compared with standard WBI in a randomized trial. Nevertheless, the high-dose treatment volume was not independently associated with an adverse cosmetic outcome. On further exploration, a V_95%_/whole-breast volume ratio <0.15 was associated with a lower risk of cosmetic deterioration (*p* = 0.04), but this accounted for a small number of the patients [20]. Although literature data seem contradictory, it seems important to preserve NTBTV as much as possible.

PTV coverage is equivalent for all treatment modalities as doses received by the homolateral lung, heart and contralateral breast. These results arise from the constraints imposed by the SHARE and NSABP protocols (Table 1). As displayed in Table 2 and Fig. 3b, the main differences between the techniques are located in the NTBTV. Non-coplanar beams may be used to reduce the proportion of NTBTV that receives high doses. It is however very unlikely that we would treat a patient using spine tracking. Using fiducials implanted inside the target volume, the CyberKnife is able to correlate the target movements to the respiratory cycle. It is thus possible to reduce PTV margins to 3 mm instead of the 10 mm that is observed without motion tracking. Using 3 mm margins, the proportion of NTBTV receiving high doses is further reduced. Differences in homolateral lung doses seem too small to be of clinical relevance. The contralateral breast also receives some dose using TOMO and CK, which is not the case using 3D-CRT. This dose remains however very low (below 2 Gy) and seems not clinically relevant in literature.

Fiducial implantation is an additional invasive process for the patients. The first part of this study shows that tracking surgical clips implanted around the resection volume during surgery is feasible (positioning accuracy below 1 mm) given that the clips are large enough to be observed on digitally reconstructed radiographs (DRR). Only the larger clips that are available (VITALITEC Medium-Large) could be tracked in our phantom.

We believe that our results may be obtained with the vast majority of patients. The phantom density was close to breast density (0.97 g.cm^−3^ as opposed to 0.95 g.cm^−3^ for adipose tissue and 1.02 g.cm^−3^ for glandular tissue). The XSight lung phantom was used to accurately reproduce the thoracic cage. To reproduce what we believe is the worst case scenario, the dimensions of our phantom were chosen to represent the largest breast size measured on 30 patients, and the target volume was positioned close to the ribs.

Our comparative dosimetric results strengthen the previously published work from Xu et al. [11] who showed the feasibility of using the CyberKnife for APBI. Their work proposed the dosimetric comparison of treatment plans calculated for 14 patients to previously published results obtained using 3D-CRT and IMRT. However, the results were somewhat limited because the authors compared their planning results to published data based on IMRT and 3D-CRT, and they did not investigate surgical clip tracking. Using different plans calculated on the same patients, we were able to compare mean DVHs for a better evaluation of the differences between techniques. To the best of our knowledge, no other study to date has compared the CyberKnife with other techniques in this manner.

For each treatment modality, dose calculations were performed using the more accurate algorithm that was available in each treatment planning station (type B, taking into account heterogeneities for secondary electron transport). For a fairer comparison, it would have been interesting to compute the dose using the same algorithm for each technique. However, we believe that the results obtained on NTBTV are too important to be a result of the dose calculation algorithm. It may also have been interesting to calculate dose distributions on 4DCTs to investigate further the benefit of tracking. Unfortunately, 4DCTs were not available for the selected patients.

In this study, we compared only the CyberKnife to tomotherapy and 3D-CRT. Comparisons between tomotherapy and 3D-CRT have previously been published [21, 22]. Because of the strict constraints imposed by the SHARE protocol, the only significant differences between tomotherapy and 3D-CRT in our work are located in the NTBTV. The direct electron field used in 3D-CRT reduces low doses to the NTBTV compared with tomotherapy.

The Cyberknife is not the only technical option to track a moving target. It would be interesting to compare the tracking accuracy of all available tracking systems and derive the CTV to PTV margins achievable with each system. However, we believe that these margins would be very close and that the results we obtained without tracking would be consistent with the results we would obtain with tracking.

## Conclusion

Tracking and non-coplanar beam directions such as used with robotic stereotactic radiotherapy in our case may be used for APBI to more efficiently spare the NTBTV. This could allow better cosmetic results. This technique could be offered to patients at a higher risk for late toxicity after APBI or in whom dosimetric constraints cannot be respected with other techniques.

## Abbreviations

3D-CRT: three dimensional conformal radiotherapy
APBI: accelerated partial breast irradiation
CK: cyberknife
CK_RM_: cyberknife with reduced margins
DSC: dice similarity coefficient
HI: homogeneity index
IMRT: intensity modulated radiotherapy
NTBTV: non target breast tissue volume
TOMO: tomotherapy
WBI: whole breast irradiation
